# Molecular Regulation of Canalicular ABC Transporters

**DOI:** 10.3390/ijms22042113

**Published:** 2021-02-20

**Authors:** Amel Ben Saad, Alix Bruneau, Elodie Mareux, Martine Lapalus, Jean-Louis Delaunay, Emmanuel Gonzales, Emmanuel Jacquemin, Tounsia Aït-Slimane, Thomas Falguières

**Affiliations:** 1 Physiopathogénèse et Traitement des Maladies du foie, Inserm, Université Paris-Saclay, UMR_S 1193, Hepatinov, 91400 Orsay, France; amel.ben-saad@inserm.fr (A.B.S.); elodie.mareux@universite-paris-saclay.fr (E.M.); martine.lapalus@universite-paris-saclay.fr (M.L.); emmanuel.gonzales@aphp.fr (E.G.); emmanuel.jacquemin@aphp.fr (E.J.); 2 Centre de Recherche Saint-Antoine (CRSA), Inserm, Sorbonne Université, UMR_S 938, Institute of Cardiometabolism and Nutrition (ICAN), 75012 Paris, France; alix.bruneau@charite.de (A.B.); jean-louis.delaunay@sorbonne-universite.fr (J.-L.D.); tounsia.ait_slimane@sorbonne-universite.fr (T.A.-S.); 3 Department of Hepatology and Gastroenterology, Charité Universitäts Medizin Berlin, 13353 Berlin, Germany; 4 Paediatric Hepatology and Paediatric Liver Transplant Department, Reference Center for Rare Paediatric Liver Diseases, FILFOIE, ERN RARE LIVER, Assistance Publique-Hôpitaux de Paris, Faculté de Médecine Paris-Saclay, CHU Bicêtre, 94270 Le Kremlin-Bicêtre, France

**Keywords:** bile secretion, ABCB1, ABCB4, ABCB11, ABCC2, ABCG5/G8, molecular partners

## Abstract

The ATP-binding cassette (ABC) transporters expressed at the canalicular membrane of hepatocytes mediate the secretion of several compounds into the bile canaliculi and therefore play a key role in bile secretion. Among these transporters, ABCB11 secretes bile acids, ABCB4 translocates phosphatidylcholine and ABCG5/G8 is responsible for cholesterol secretion, while ABCB1 and ABCC2 transport a variety of drugs and other compounds. The dysfunction of these transporters leads to severe, rare, evolutionary biliary diseases. The development of new therapies for patients with these diseases requires a deep understanding of the biology of these transporters. In this review, we report the current knowledge regarding the regulation of canalicular ABC transporters’ folding, trafficking, membrane stability and function, and we highlight the role of molecular partners in these regulating mechanisms.

## 1. Introduction

**Figure 1:**
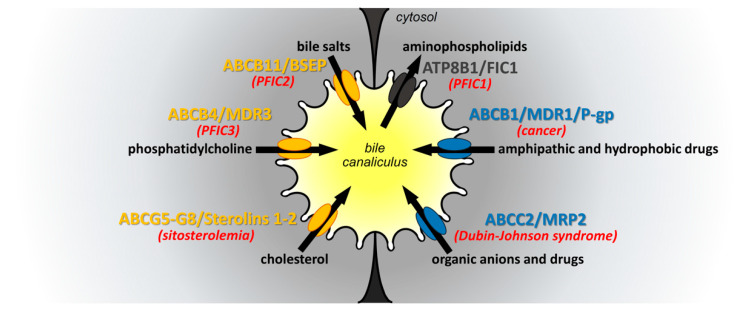
ATP-binding cassette (ABC) transporters at the canalicular membrane of hepatocytes. The bile canaliculus is formed by the canalicular membrane of hepatocytes. The main canalicular ABC transporters are indicated according to the nature of their substrates: in yellow for hydrophobic substrates and in blue for drugs. Note that ATP8B1 is not an ABC transporter but a P-type ATPase. However, its function is tightly related to the other canalicular ABC transporters. The substrates of these transporters are shown in black, and their flows are indicated by black arrows. The main diseases associated with functional defects of these transporters are indicated in red. PFIC: progressive familial intrahepatic cholestasis.

One of the liver’s main functions is bile production and secretion. In addition to its digestive function, bile plays an important role in detoxification. Bile secretion is mediated by several ATP-binding cassette (ABC) transporters, which are expressed at the canalicular membrane of hepatocytes. The main canalicular ABC transporters are the bile salt export pump (BSEP, ABCB11), which transports bile acids (BAs), ABCB4 also known as multidrug resistance protein 3 (MDR3) translocating phosphatidylcholine (PC) and ABCG5/G8 excreting cholesterol [1]. BA, PC and cholesterol form mixed micelles in the aqueous environment of bile. In addition to these compounds, bile contains a wide variety of drugs and organic anions, which are secreted by ABCB1, also known as multidrug resistance protein 1 (MDR-1, or P-glycoprotein) and ABCC2 (multidrug resistance-associated protein 2, MRP2), respectively [2,3]. ABC transporters share a common basic architecture and similar ATP-driven functions. They are organized in two repeats, each containing a membrane-spanning domain (MSD) with six transmembrane (TM) helices and a cytoplasmic nucleotide-binding domain (NBD), those two moieties being connected by an intracellular linker. The MSDs ensure substrate recognition and translocation, whereas NBDs, which are highly conserved among all ABC transporters, provide the energy for this process [3]. In contrast to other canalicular ABC transporters, ABCG5 and ABCG8 are half transporters that require heterodimerization to ensure their function, and ABCC2 has a third MSD at its N-terminus [4]. The key role of canalicular ABC transporters in bile secretion is highlighted by their implication in a wide range of diseases such as cholestasis (ABCB4, ABCB11), sitosterolemia (ABCG5/G8), Dubin–Johnson syndrome (ABCC2) and cancer (ABCB1) (Figure 1) [3]. To develop new therapies for patients with diseases related to deficient canalicular ABC transporters, it is crucial to better understand the molecular mechanisms regulating the traffic and function of these transporters. Several studies have reported that the biosynthesis, trafficking and activity of ABC transporters are regulated by numerous molecular partners, most of which have been identified by two-hybrid screens using liver banks [3]. Targeting these interactors represents a potential therapeutic option for patients. This review focuses on molecular regulators of canalicular ABC transporters involved in bile formation.

## 2. Folding and Glycosylation of Canalicular ABC Transporters

Protein folding is a highly regulated process that is mediated by numerous factors, including folding proteins and molecular chaperones [5]. Some of these proteins have been shown as interactors of canalicular ABC transporters, controlling their biosynthesis and folding. As most transmembrane proteins, ABC transporter biosynthesis starts with their cotranslational translocation and insertion into the endoplasmic reticulum (ER) membrane through the Sec61 translocon complex [6]. Numerous accessory factors were described to facilitate the translocation process, including the translocating chain-associated membrane protein (TRAM) and the translocon-associated protein (TRAP) [7]. Interestingly, TRAM and three subunits of the TRAP complex (SSR1, SSR3 and SSR4) were found to interact and coprecipitate specifically with ABCB11 [8].

Once in the ER lumen, nascent ABC transporters are N-glycosylated. Glycans are added to their extracellular asparagine residues [9] and play a critical role in protein folding, stability and interaction with some chaperones [10]. Two main chaperone families exist in the ER: the heat shock protein (HSP) family, which promotes the folding of a wide variety of proteins, and the lectin chaperones, which recognize and fold specifically glycosylated proteins [11]. Calnexin (CNX) and its soluble homolog calreticulin are lectin chaperones that were shown to bind both ABCG5 and ABCG8 and stimulate their folding and assembly. Okiyoneda and colleagues showed that the silencing of either CNX or calreticulin decreases the expression of ABCG5/G8 [12]. It has also been reported that CNX and the heat shock cognate 71 kDa protein (Hsc70) interact with ABCB1 [13,14].

In addition to their folding function, chaperones are central players in the quality control process. They evaluate the folding state of proteins and regulate their ER retention or ER exit [15]. They allow only properly folded proteins to exit the ER, and contrariwise, they retain abnormally folded proteins longer before their targeting to the ER-associated degradation (ERAD) pathway. Some variations in canalicular ABC transporter-encoding genes were described to affect the folding of these transporters, thereby leading to their retention in the ER. Indeed, a prolonged association between misfolded ABCB1 variants and CNX has been observed [13]. Interestingly, we and others have demonstrated that several small molecules known as pharmacological and chemical chaperones can facilitate the folding and exit of defective ABCB4 and ABCB11 variants from the ER [16,17,18]. Another key component of the quality control system is the B-lymphocyte receptor-associated protein (BAP), which was shown to control the folding state and sorting of many proteins in the ER [19,20]. The BAP29 and BAP31 isoforms were described to interact with the N-terminal domain of ABCB1 and ABCB11, respectively [8,21]. More interestingly, it has been shown that some mutations in the *BAP31* gene are associated with liver dysfunction and cholestasis [22].

Using an immunoprecipitation assay combined with mass spectrometry analysis and yeast two-hybrid screens, Przybylla and colleagues identified several novel potential ER-resident partners for ABCB11, including the receptor expression-enhancing proteins (REEPs) involved in ER shaping, the immediate early response 3 interacting protein 1 (IER3IP1), the transmembrane proteins 205 (TMEM205) and 14A (TMEM14A) and the bile Acyl-CoA synthetase (BAC) [8]. However, their role in the regulation of the folding and/or the trafficking of ABCB11 has not been studied yet.

## 3. From the Endoplasmic Reticulum to the Plasma Membrane

Correctly folded canalicular ABC transporters leave the ER to reach the Golgi apparatus, where they undergo further post-translational modifications. However, little is known about the molecular players regulating their trafficking from the ER to the Golgi. Involvement of the coat protein complex II (COP II) machinery in ABCC7/cystic fibrosis transmembrane conductance regulator (CFTR) and ABCA1 exit from the ER has been documented [23,24]. Given the homology between these proteins, the same pathway may be involved in the sorting and traffic of canalicular ABC transporters. Once in the Golgi apparatus, canalicular ABC transporters undergo more complex glycosylation [25,26]; then, they are sorted and packaged into secretory vesicles and further delivered to the canalicular membrane [25,26].

Unlike other apical proteins in liver cells, canalicular ABC transporters do not undergo transcytosis after their sorting from the trans-Golgi network (TGN), but they are directly targeted to the canalicular membrane or subapical compartments (SACs) [27,28]. The labeling of newly synthesized ABC transporters has shown that ABCB1 is directly delivered to the canalicular membrane, whereas ABCB11 is targeted to the SAC before reaching the canalicular membrane [29]. Kipp and coworkers also described the involvement of many intracellular components, such as cyclic adenosine monophosphate (cAMP), taurocholate and Ca^2+^ in the vesicular trafficking of canalicular ABC transporters. Indeed, they showed that the administration of these components into the perfused liver or directly in cells increases the amount of ABC transporters present at the canalicular membrane as well as bile secretion [28].

In addition to these components, many interacting proteins, including specific GTPases, kinases, molecular motors and other factors, have been shown to associate with canalicular ABC transporters and promote their exocytosis and/or endocytosis. Indeed, CFTR-associated ligand (CAL), a Golgi-associated protein, has been found to interact with ABCC2 and regulate its plasma membrane targeting [30]. Some members of the Ras-related in brain (RAB) GTPase family have also been identified as ABCB1-interacting proteins. The overexpression of RAB4, RAB5 or their constitutively active forms increases the presence of ABCB1 at the cell surface [31,32]. Moreover, the motor protein myosin II regulatory light chain (MLC2) was reported as a prominent regulator of canalicular ABC transporters. Using a yeast two-hybrid screen of a rat liver cDNA library, MLC2 was found to interact with the linker domains of ABCB1, ABCB4 and ABCB11 [33]. Based on immunofluorescence and biochemical experiments, Chan and colleagues showed that the inhibition of MLC2 or the expression of its dominant negative form leads to a decrease in ABCB11 levels at the apical membrane [33].

Furthermore, other studies have revealed that many kinases are important for the exocytosis of ABC transporters. These include the p38 mitogen-activated protein kinase (MAPK) [34,35], protein kinase A (PKA [36], protein kinase C (PKC) [37], proto-oncogene serine/threonine-protein kinase (Pim-1) [38] and phosphoinositide 3-kinase (PI3K) [39]. Misra and coworkers showed that the administration of wortmannin, a specific inhibitor of PI3K, resulted in a decrease in the amounts of ABCB11 and ABCC2 present at the canalicular membrane [39,40]. Another kinase, the liver kinase B1 (LKB1), was shown as a key regulator of ABCB11 trafficking. In LKB1 knockout (KO) mice, an altered distribution of ABCB11, as well as an impaired bile formation, was observed [41,42]. Ursodeoxycholic acid (UDCA), used as a treatment for patients with cholestasis, was also shown to stimulate the targeting of ABCC2 and ABCB11 transporters to the plasma membrane [43,44].

## 4. Membrane Stability of Canalicular ABC Transporters

Membrane protein turnover through uninterrupted synthesis and degradation is essential to provide a functional set of proteins and ensure cell function. Tight regulation of protein stability at the plasma membrane is fundamental for cell homeostasis and relies on environmental signals and/or post-translational modifications such as phosphorylation/dephosphorylation and ubiquitination/deubiquitination cycles. Indeed, on the one hand, the accumulation of some proteins at the plasma membrane can be deleterious for cells and result, for instance, in a multidrug resistance (MDR) phenotype, a true obstacle in cancer treatment, caused by the development of chemoresistance [45]. On the other hand, defects in the expression level or stability of ABC transporters can also contribute to the development of human diseases, including cystic fibrosis [46], neuropathies [47] or cholestasis [48]. The regulation of the stability/turnover of proteins such as canalicular ABC transporters remains poorly understood, mostly due to technical limitations. ABC transporter stability is yet essential to regulate the spatiotemporal availability of a given protein at the bile canaliculi (e.g., between meals, the need for bile is reduced, and the amount of ABC transporters at the plasma membrane must be regulated accordingly). On the contrary, a decrease in the stability of numerous transporters at the plasma membrane, such as ABCB1, ABCC1 (MRP1) or ABCG2 (BCRP), would be necessary to improve the efficiency of cancer treatments facing ABC transporter-mediated MDR.

Several kinases are involved in the regulation of ABC transporter stability. The atypical Pim-1 kinase coimmunoprecipitates with and phosphorylates ABCB1. Pim-1 downregulation by siRNA diminishes ABCB1 maturation and favors its degradation through the ubiquitin–proteasome system, indicating that Pim-1 may stabilize ABCB1 at the plasma membrane [38]. A yeast two-hybrid screen using the linker domain of ABCB4 allowed the identification of receptor for activated C-kinase 1 (RACK1) as an interacting partner of this transporter. Moreover, RACK1 has been reported to activate two isoforms of PKC and be involved in the regulation of membrane stability for many proteins, thus playing a determinant role in fundamental cellular activities [49]. Following RACK1 knockdown, ABCB4 is no longer localized at the plasma membrane but mainly relocalized in cytosolic compartments [50].

PDZ (postsynaptic density protein (*PSD95*), Drosophila disc large tumor suppressor (*Dlg1*) and zonula occludens-1 protein (*ZO-1*))-domain-containing proteins are well known for their function in protein stabilization at membranes. They act as scaffolds by linking transmembrane proteins to the cytoskeleton and thus regulate their subcellular localization and stability at the plasma membrane [51]. The PDZ-domain-containing protein ezrin–radixin–moesin (ERM)-binding phosphoprotein 50 (EBP50), also known as sodium–hydrogen exchanger regulatory factor-1 (NHERF1), interacts with both ABCC2 and ABCB4 through their C-terminal PDZ-binding motif [52,53]. In the absence of EBP50, ABCB4 and ABCC2 are both targeted to the plasma membrane, but their presence is drastically reduced, therefore demonstrating that EBP50 plays a crucial role in the regulation of membrane stability for both ABCC2 and ABCB4 [52,53]. PDZK1 (NHERF3), another PDZ domain-containing protein, interacts with ABCC2 and increases its plasma membrane stability. Indeed, the expression of a dominant negative form of PDZK1 leads to a decrease in ABCC2 membrane expression and its accumulation in intracellular compartments [54,55].

Radixin is part of the ERM protein family, which is involved in actin cytoskeleton remodeling, e.g., to organize submembranous cortical actin or microvillosities [56]. Radixin KO mice develop a phenotype comparable to Dubin–Johnson syndrome. Indeed, these mice show a severe reduction in ABCC2 protein expression at bile the canaliculi without any change at the mRNA level. Importantly, this effect is specific to ABCC2 as no effect was observed for other canalicular ABC transporters such as ABCB1, ABCB11 or ABCB4 [57]. Moreover, a direct interaction between ABCC2 and radixin has been confirmed by GST-pulldown [57].

## 5. Endocytosis and Membrane Recycling of Canalicular ABC Transporters

Small GTPases, protein kinases, class V myosins and adaptor proteins have been identified as molecular players in the regulation of canalicular ABC transporter endocytosis and recycling [3]. The existence of ABCB11 intracytoplasmic reservoirs is well known [28,29], but the nature of those compartments long remained uncharacterized until a YFP-tagged ABCB11 was detected in the SAC, a RAB11-positive compartment. Indeed, RAB11 and myosin VB (MYO5B) are established regulators of the recycling of several proteins from the SAC to the plasma membrane [58]. ABCB11 continuously cycles between the canalicular membrane of hepatocytes and the SAC [59]. This constant exchange allows tight regulation of ABCB11 availability at the bile canaliculi. The perturbation of actin cytoskeleton or microtubules inhibits this traffic [59]. These results were corroborated as ABCB11 apical targeting is considerably slowed down in WIF-B9 cells expressing RAB11- or MYO5B-dominant negative constructs [60]. In the presence of a mutated or truncated MYO5B, ABCC2 displays an intracellular localization in RAB8- and RAB11-positive compartments, suggesting defects in canalicular transporter recycling [61]. Recently, mutations in the *MYO5B* gene, identified in patients, have been associated with a progressive familial intrahepatic cholestasis (PFIC)-like phenotype, further proposed as PFIC6 [61,62].

The ERM protein family has also been involved in the endocytic process of several ABC transporters. Coimmunoprecipitation performed with human liver lysates highlighted the interaction between ABCC2 and ezrin, and additional experiments showed that ezrin phosphorylation on its Thr567 regulates this interaction, thus further controlling the amounts of ABCC2 present at the plasma membrane [63].

Several isoforms of PKC, as well as PKA, PI3K, Pim-1 or Fyn kinases, play a role in the regulation of ABCB1, ABCC2 and ABCB11 membrane targeting or endocytosis [33,39,41,42,64,65,66]. Cantore and colleagues have reported that the Src family kinase Fyn induces ABCC2 and ABCB11 retrieval from the canalicular membrane by increasing cortactin phosphorylation [66]. Schonhoff and colleagues observed that taurolithocholate-activated PKCε phosphorylates and activates myristoylated alanine-rich C-kinase substrate (MARCKS), a membrane-bound F-actin crosslinking protein [67]. MARCKS is a crucial regulator of molecular interactions and cytoskeletal reorganization. In a nonphosphorylated state, MARCKS is associated with the cytosolic leaflet of the plasma membrane and can serve as a stabilizer for transmembrane proteins, whereas after phosphorylation, MARCKS is released in the cytosol, where it can interact with other proteins [68].. MARCKS has been shown to regulate the endocytosis of ABCC2 and ABCB1 [67,69]. Indeed, in colon carcinoma cells, MARCKS expression has been associated with the reduced export function of ABCB1 [69].

The hematopoietic cell-specific Lyn substrate 1 associated protein X-1 (HAX-1) is a small protein abundantly expressed in the liver, regulating cortical actin organization. This protein has been identified as an interactor of the linker domain of ABCB1, ABCB4 and ABCB11 [70]. Through this interaction, HAX-1 has been proposed to stabilize ABCB11 at the plasma membrane [70]. However, the role of HAX-1 in other canalicular ABC transporter endocytosis has not been further investigated.

A tyrosine motif has been identified in the ABCB11 cytoplasmic tail [71] along with one of its partners, the clathrin adaptor protein complex 2 (AP2) [72]. AP2 is localized at the plasma membrane and binds tyrosine-based internalization motifs of proteins, including ABCB11, thus allowing its internalization from the canalicular membrane through clathrin-dependent endocytosis [72]. The ubiquitination of ABCB11 and ABCC2 has also been shown to be essential for clathrin-mediated endocytosis and degradation of these transporters [73].

Hormones and intracellular signaling molecules also play a role in canalicular ABC transporter internalization. In a model of estradiol-induced cholestasis, authors showed that following treatment with estradiol-17β-d-glucuronide (E217G), ABCB11 and ABCC2 are relocalized from canalicular membranes to intracytoplasmic compartments. The same group demonstrated later that ABCB11 and ABCC2 endocytosis is mediated by PKC, which is activated by E217G, and that PKC inhibitors prevent the internalization of both transporters after treatment with estradiol [74,75]. In the same model, Zuchetti and colleagues established that glucagon and an adrenaline analog mediate cAMP activation, thus preventing ABCB11 and ABCC2 membrane retrieval [76]. Moreover, they showed that this E217G-induced endocytosis is AP2- and clathrin-dependent [77]. It has also been suggested that lipopolysaccharides act as signals for ABCC2 and ABCB11 endocytosis as their canalicular expression is reduced, with no mRNA decrease in an in vitro cholestatic model [78].

## 6. Regulation of the Transport Activity of Canalicular ABC Transporters

At the canalicular membrane, ABCB1, ABCB4, ABCB11, ABCC2 and ABCG5 have been proposed to mostly reside within glycosphingolipids-, cholesterol- and caveolin-1 (Cav-1)-enriched raft microdomains [27,79,80,81]. These domains could provide a favorable environment for the regulation of the activity of canalicular ABC transporters. Indeed, the shift of ABCB1 and ABCB11 from cholesterol-enriched microdomains to low-cholesterol domains lowered their transport activity [82,83,84,85]. Moreover, accumulating evidence indicates that phospholipids and cholesterol are required for the proper function of ABCB1, ABCB4, ABCB11 and ABCC2 [86,87,88,89,90,91,92,93]. In purified membrane vesicles, delipidation due to detergent action inactivates ABCB1, whereas phospholipid addition fully restores the ATPase activity of the transporter [94,95]. Phosphoinositides, lipid products from PI3K-mediated activities, are required for ABCB11 and ABCC2 activation because their addition reverses the negative effect of PI3K inhibitors on the activity of these transporters [40,96].

The importance of membrane cholesterol content has been highlighted in Atp8b1-deficient mice [92]. Indeed, in these mice, the normal phospholipid asymmetry of the canalicular membrane is lost, thereby enhancing sensitivity to cholesterol extraction by hydrophobic BA and subsequent loss of ABCB11 and ABCC2 activity [92]. How exactly membrane cholesterol influences the transport activity of these transporters is not known, but this may involve allosteric modulations and/or indirect means such as changes in membrane fluidity. Several studies have proposed that cholesterol directly interacts with the ABCB1 substrate binding site and thereby facilitates the recognition of small drugs (<500 Da) [89,97,98]. Cyclodextrin treatment or Cav-1 overexpression leads to cholesterol depletion from the plasma membrane, which inhibits ABCB1 transport activity by increasing membrane fluidity and loosening lipid packing density [99]. However, Moreno and colleagues showed that Cav-1 overexpression in mice increases both bile flow and the biliary secretion of phospholipids, BA and cholesterol, suggesting a positive role of Cav-1 in ABCB11 transport activity [100]. Considering the role of Cav-1 in intracellular cholesterol trafficking [101,102], some of the Cav-1 effects may be indirect and mediated through cholesterol homeostasis. Alternatively, Cav-1 may also directly bind ABCB1 and inhibit its transport activity [99,103,104,105]. It has been reported that the binding capacity of Cav-1 to ABCB1 is negatively modulated by Src kinase-mediated Cav-1 phosphorylation, a process facilitated by RACK1, which interacts with both Src and ABCB1 [105,106,107].

Several studies suggest a role for phosphorylation in the regulation of canalicular ABC transporters. Phosphorylation sites in the linker domain of ABCB1 have been well documented at Ser661, Ser667, Ser671, Ser675, and Ser683 [108,109,110,111,112]. Likewise, six potential phosphorylation sites have been found in the linker region of ABCC2 at Ser904, Ser912, Ser916, Ser917, Ser922 and Ser926 [113] and in the N-terminal domain of ABCB4 at Thr34, Thr44 and Ser49 [114]. An analysis of ABCB11 amino acid sequence also predicted multiple potential serine/threonine phosphorylation sites [115]. Overwhelming evidence indicates that PKC is a major player in ABC transporter phosphorylation and activity regulation. Phosphorylation within the linker domain of ABCB1 is specific for PKCα in purified vesicles from Sf9 cells [116]. The coexpression of PKCα and ABCB1 increases the ATPase activity of the transporter in insect and ovarian cells [116,117], while PKC inhibitor treatment did not alter ATPase activity in MCF-7 cells [118]. It has been shown that PKCα also mediates ABCB11 phosphorylation [115], as well as ABCC2 phosphorylation, resulting in stimulation of the intrinsic transport activity of ABCC2 [113]. PKC-dependent phosphorylation has also been shown to regulate ABCB4-mediated PC secretion [114]. The variation-induced impairment of ABCB4 N-terminal phosphorylation may also be related to a decrease in ABCB4-mediated PC secretion [114]. The substitution of all conserved serines in the linker domain of ABCC2 by non-phosphorylatable alanines significantly reduces the basal transport activity of ABCC2, while substitution into aspartates (mimicking constitutive phosphorylation of the residues) increases it [113]. Conversely, the role of ABCB1 phosphorylation in the regulation of its transport activity is less obvious because the substitution of potentially phosphorylatable residues by aspartates or non-phosphorylatable residues has no effect [119,120].

## 7. Ubiquitination and Degradation of Canalicular ABC Transporters

To target proteins for degradation, cells mostly use the endolysosomal pathway vs. proteasomal degradation, related to the monoubiquitination or polyubiquitination of their substrates, respectively [121,122].

The lysosomal pathway is the main way by which cells turn over plasma membrane proteins. Indeed, ABCB1 colocalizes with lysosomal-associated membrane protein 1 (LAMP1) in human colorectal cancer HTC15 cells [123]. In addition, the half-life of ABCB1 and ABCC2 is extended in cells treated with lysosomal inhibitors alone but not proteasomal inhibitors alone, suggesting the involvement of the lysosomal pathway in the degradation of these transporters [123,124]. However, ABCB11 expression is unaffected by treatment with lysosomal inhibitors, indicating that this transporter may use another degradation pathway [125,126]. Indeed, it has been shown that the inhibition of proteasomal degradation stabilizes wild-type (WT) and mutated ABCB11 in MDCK and HEK cells, suggesting that ABCB11 degradation involves the proteasome [125,127].

Several E3 ubiquitin ligases (E3 Ubl) may be involved in canalicular ABC transporter degradation. Ring finger protein 2 (RNF2) has E3 Ubl activity and may mediate the ubiquitination of ABCB1 [128]. E3 Ubl FBXO21 is involved in the proteasome-mediated degradation of ABCB1 [129]. Additionally, the E2-conjugating enzyme UBE2R1 (also named CDC34 or UBC3) and the E3 complex Skp1–Cullin–FBOX15 (SCFFbx15) are both implicated in ABCB1 ubiquitination [130]. Coprecipitation assays revealed that FBX015/Fbx15 (a member of the SCFFbx15 E3 complex) and UBE2R1 both interact with ABCB1, and their knockdown is associated with a decrease in ubiquitination and subsequent degradation of ABCB1. By contrast, FBX015 expression enhances ABCB1 ubiquitination and degradation [130].

ABCB1 ubiquitination may be modulated by the MAPK pathway [131,132]. Indeed, the inhibition of MEK or the downregulation of its downstream effectors, such as ERK and p90 ribosomal S6 kinases (RSKs), lower ABCB1 protein expression in HTC15 cells [131,133]. Pulse-chase labeling experiments revealed that MEK inhibitor-mediated downregulation of ABCB1 is caused by the increase of its degradation [131]. The same team has shown that RSK1 induces self-ubiquitination of UBE2R1, followed by its proteasomal degradation in a phosphorylation-dependent manner, thus resulting in the protection of ABCB1 against degradation [132].

Some variations in ABC transporter genes are responsible for the production of an unstable protein which is retained in the ER and subsequently degraded in the cytosol by the ERAD system [134]. Some misfolded ABCB11 variants appear to be more ubiquitinated than the WT transporter [126]. The RING finger proteins Rma1, TEB4 and HRD1 are all E3 Ubl involved in the ubiquitination of ABCB11-WT and its variants but with a folding sensitivity as the knockdown of each E3 Ubl stabilizes different ABCB11 variants [126]. HRD1 targets proteins with defects in the ER lumen side, while TEB4 and Rma1 target proteins with defects in their moieties facing the cytosol. Likewise, E3 Ubl seems to exhibit sensitivity towards ABC transporters. GP78, rather than TEB4 and HDR1, plays an important role in the ubiquitination of ABCC2, as shown in patients with obstructive cholestasis and in rifampicin-treated HepG2 cells [63,135]. Proteins can escape ubiquitination through small ubiquitin-like modifier (SUMO) modification as both processes compete on the same residues. Using a protein–protein interaction assay, a number of SUMO-related proteins (including SUMO-1 and ubiquitin carrier protein 9/Ubc9) were pulled down using the linker region of the rat ABCC2 [136]. Moreover, the knockdown of SUMO-related enzymes in hepatoma cells reduces ABCC2 protein expression but not its mRNA expression or canalicular localization [136]. Proteins can escape degradation subsequent to their ubiquitination by reversing ubiquitination thanks to deubiquitinating enzymes (DUBs). As an example, the DUB ubiquitin-specific protease 19 (USP19), through TEB4 stabilization, negatively regulates the expression of a defective ABCB11 variant [137].

Manipulation of the ER quality control system might be combined with chemical or pharmacological chaperones to stabilize variants and restore the cell surface expression of ABC transporters. Indeed, cell surface biotinylation assays revealed that the most frequent ABCB11 variants found in patients with PFIC2, E297G and D482G are highly ubiquitinated [138], and this induces their internalization [73]. Additionally, the half-life of ABCC2 is extended in cells overexpressing a dominant negative form of ubiquitin due to the inhibition of ABCC2 degradation [73,124]. Therefore, by reducing susceptibility to ubiquitination, the chemical chaperone 4-phenylbutyrate (4-PB) extends the half-life of both ABCB11 and ABCC2 expressed at the cell surface [124,138,139]. However, since 4-PB has no effect on ABCB1 [139], the 4-PB mechanism of action would involve interaction with specific E3 Ubl or adaptor protein(s) for both ABCB11 and ABCC2. For instance, 4-PB downregulates Hsc70 (Hsp73) which plays a role in the lysosomal degradation of intracellular proteins and was shown to be required for the ubiquitin-dependent degradation of several proteins ([140]. and references therein).

## 8. Conclusions

Over the last decade, proteomic studies have become an important means for understanding the biology and pathophysiology of many proteins. The characterization and identification of key players prompted the understanding of the molecular basis of pathologies and helped the development of improved therapeutic approaches for patients. We described here the molecular partners that interact either directly or indirectly with the five canalicular ABC transporters (ABCB11, ABCB4, ABCG5/G8, ABCB1 and ABCC2) and regulate their folding, trafficking, stability and function (Table 1). Nowadays, an important amount of information regarding the genetics of ABC transporters is gathered. We expect that proteomic approaches merged with genomic studies will be a powerful tool in the development of personalized treatment for patients with biliary diseases related to canalicular ABC transporter defects.

## Figures and Tables

**Table 1:** Molecular partners of canalicular ABC transporters.

Proteins ^1^	Interacting ABC Transporters	Subcellular Localization	Functions	References
AP2	ABCB11	Plasma membrane	Clathrin-dependent endocytosis	[71,72,73,77]
BACs	ABCB11	ER	Conjugation of bile acids	[8]
BAP29	ABCB1	ER	Controls protein sorting from the ER	[21]
BAP31	ABCB11	ER	Controls protein sorting from the ER	[8]
CAL	ABCC2	Golgi	Golgi sorting	[30]
Calnexin	ABCG5/G8 ABCB1	ER	Assists glycoprotein folding	[12,13,16]
Calreticulin	ABCG5/G8	ER	Assists glycoprotein folding	[12]
Cav-1	ABCB1 ABCB4 ABCB11 ABCC2 ABCG5/G8	Plasma membrane	Scaffold protein	[99,100,103,104,105,107]
CD44	ABCB1	Plasma membrane	Inhibitor of FBX021	[129]
EBP50	ABCB4 ABCC2	Plasma membrane	Scaffold protein	[52,53]
Ezrin	ABCC2	Plasma membrane Associated with the cytoskeleton	Endocytosis	[63]
FBXO21	ABCB1	Cytosol	E3 ubiquitin ligase	[129]
Fyn	ABCC2 ABCB11	Plasma membrane	Endocytosis	[66]
GP78	ABCC2	ER	SUMO-related proteins	[63,135]
HAX-1	ABCB1 ABCB4 ABCB11	Cytosol Associated with cortical actin	Clathrin-dependent endocytosis	[70]
Hsc70	ABCB1	ER	Chaperone Assists protein folding	[14,16]
IER3IP1	ABCB11	ER	Implicated in apoptosis and protein transport from the ER to the Golgi	[8]
LKB1	ABCB11	Cytoplasm	Intracellular traffic	[41,42]
MARCKS	ABCC2 ABCB1	Cytosol Plasma membrane	Endocytosis	[67]
Myosin Vb	ABCB11	Cytosol Recycling endosomes Plasma membrane	Recycling to the plasma membrane	[60]
MLC2	ABCB1 ABCB4 ABCB11	Cytosol	Motor protein	[33]
PDZK1	ABCC2	Cytosol	Promotes membrane stability	[54,55]
Pim-1	ABCB1	Cytosol	Promotes membrane stability	[38]
PI3K	ABCB4 ABCB11 ABCC2	Plasma membrane Cytosol	Protein kinase	[39]
PKA and PKC	ABCB1 ABCB11 ABCC2	Plasma membrane	Protein kinase	[113,114,115,116,117,118]
RAB4	ABCB1	Endosomes Plasma membrane Cytosol	Vesicular trafficking	[31]
RAB5	ABCB1	Endosomes Plasma membrane Cytosol	Vesicular trafficking	[32]
RAB8	ABCC2	Endosomes Plasma membrane Cytosol	Vesicular trafficking	[61]
RAB11	ABCB11	Endosomes Plasma membrane Cytosol	Vesicular trafficking	[60]
RACK1	ABCB1 ABCB4	Plasma membrane	Scaffold protein	[107]
Radixin	ABCC2	Cytosol Plasma membrane	Promotes membrane stability	[57]
REEP	ABCB11	ER	ER shaping and remodeling	[8]
Rma1, TEB4 and HRD1	ABCB11	ER	E3 ubiquitin ligases	[126]
RNF2	ABCB1	Cytosol	E3 ubiquitin ligase	[128]
RSK1	ABCB1	Cytosol	Kinase	[131,133]
SCFFbx15	ABCB1	Cytosol	E3 ubiquitin ligase	[130]
Src kinase	ABCB1	Plasma membrane	Protein kinase	[105,106,107]
TMEM14A	ABCB11	ER	Implicated in apoptosis	[8]
TMEM205	ABCB11	ER	Drug resistance	[8]
TRAM/TRAP	ABCB11	ER	Accessory protein in the Sec61 translocon complex	[8]
UBC9	ABCC2	Cytosol	SUMO-related protein	[136]
UBE2R1	ABCB1	Cytosol	E2 ubiquitin-conjugating enzyme	[130,132]
USP19	ABCB11	ER	Deubiquitinating enzyme	[137]

^1^ See the main text for full names of the proteins.

## Data Availability

Not applicable.

## Abbreviations

4-PB	4-phenylbutyrate
ABC	ATP-binding cassette
AP2	Adaptor protein complex 2
BA	Bile acids
Cav-1	Caveolin-1
CNX	Calnexin
E217G	Estradiol-17β-d-glucuronide
E3 Ubl	E3 ubiquitin ligase
ER	Endoplasmic reticulum
ERM	Ezrin–radixin–moesin
ERAD	ER-associated degradation
MARCKS	Myristoylated alanine-rich C-kinase substrate
MLC2	Myosin regulatory light chain 2
PC	Phosphatidylcholine
PDZ	Postsynaptic density protein (PSD95), Drosophila disc large tumor suppressor (Dlg1) and zonula occludens-1 protein (ZO-1)
PFIC	Progressive familial intrahepatic cholestasis
PI3K	Phosphoinositide 3-kinase
PKA/C	Protein kinase A/C
RAB	Ras-related in brain
RACK1	Receptor for activated C-kinase 1
SAC	Subapical compartment
WT	Wild type
